# Methods to Engage Community Partners for Research to Increase Social Connection Among at-Risk Older Adults

**DOI:** 10.1093/geroni/igaf122.858

**Published:** 2025-12-31

**Authors:** Jin hui Joo, Ryan Mace, Rebekka Lee, Namkee Choi, Evan Plys, Nancy Donovan, Joseph Locascio

**Affiliations:** Massachusetts General Hospital, Boston, Massachusetts, United States; Massachusetts General Hospital, Boston, Massachusetts, United States; Harvard University, Cambridge, Massachusetts, United States; The University of Texas at Austin, Austin, Texas, United States; Massachusetts General Hospital, Boston, Massachusetts, United States; Harvard Medical School, Boston, Massachusetts, United States; Massachusetts General Hospital, Boston, Massachusetts, United States

## Abstract

Social isolation and loneliness are critical public health issues in the U.S. and worldwide, with studies reporting a prevalence ranging from 22% to 61% of adults who feel lonely. Loneliness puts a person at risk for multiple medical and mental health problems such as cardiovascular disease, depression, diabetes, dementia as well as increased the risk of all cause mortality. Systematic reviews have concluded that established interventions, cognitive behavioral therapy (CBT, addressing unhelpful social cognitions and behaviors) and Social Prescribing (SP, linking to non-clinical community-based resources). We partnered with the Massachusetts Council on Aging and the Cambridge Housing Authority to develop a comparative effectiveness RCT PCORI proposal of these two interventions. In the process, we used multiple strategies to engage our community partners. These strategies included: ongoing engagement over 12 months, numerous in person and virtual listening sessions with community members ranging from older adults, organization staff and leaders, and scientific experts, attention to building capacity for research through knowledge exchange, built in processes and organizational structure to integrate fully various community partners with complementary expertise and experience, and finally ongoing assessment of engagement throughout the project. Numerous discussions and listening sessions were held with elders, leaders, and frontline staff at senior centers and public housing organizations. The result was bidirectional knowledge about CBO (community-based organization) priorities, needs, resources and processes “on the ground” as well as research principles and process that shaped the research design and established an ongoing foundation of trust and rapport.

